# Hun Surgeon's Murder

**Published:** 1917-11-10

**Authors:** 


### HUN SURGEON'S MURDER.

### Fatal Operation for Prince's Whim.

WE reprint the whole of this from the *Daily Mail* of the 2nd instant:—

AMSTERDAM, Thursday.

Disciplinary proceedings have been taken against Dr. Max Henkel, a professor of gynæcology of the Jena Women's Clinic, respecting charges against him in connection with his practice. The case ended yesterday in judgment being given ordering his removal from his position as director of the Jena University Women's Clinic and condemning him to pay the costs.

Henkel among other things admitted Prince zu Lippe to his operating theatre to see an operation, and in view of the presence of his exalted guest a case intended for an operation at a later date was taken earlier. The German papers publish the following evidence of the assistant physician concerning this proceeding:—

"A civilian suddenly entered the operating-room, and Henkel asked Professor Busse, 'Is there nothing more to operate on?' Busse replied in the negative, whereupon Henkel rejoined, 'But we must operate on something else!' Busse said there was nothing else. Henkel replied, 'You have one more case; bring it in!' Busse said that the patient, a woman, had just breakfasted, but Henkel said, 'It doesn't matter.'

"The case was then prepared. The woman was very much excited that she should be so suddenly operated upon, and a very disagreeable scene followed. She was very quickly operated on, and all appeared to go off very well. Suddenly a nurse plucked my coat, and I saw that the woman lay *in extremis*. I gave her a couple of camphor injections, but she died. I went and told Henkel, who nodded silently.

"Prince zu Lippe said to Henkel: 'You operated splendidly. I must tell my sister about it immediately.'

"I had an instinctive feeling that if the prince had known the tragic issue he would have spoken otherwise. I spoke about the proceeding to another assistant physician, who in reply said something which completely knocked me over: 'But Henkel is the greatest criminal!'"

